# Long QT syndrome: A therapeutic challenge

**DOI:** 10.4103/0974-2069.41051

**Published:** 2008

**Authors:** Maully Shah, Christopher Carter

**Affiliations:** The Cardiac Center, The Children's Hospital of Philadelphia, Philadelphia, USA

**Keywords:** Long QT syndrome, torsade de pointes

## Abstract

Congenital long QT syndrome (LQTS) is one of the most common cardiac channelopathies and is characterized by prolonged ventricular repolarization and life-threatening arrhythmias. The mortality is high among untreated patients. The identification of several LQTS genes has had a major impact on the management strategy for both patients and family members. An impressive genotype-phenotype correlation has been noted and genotype identification has enabled genotype specific therapies. Beta blockers continue to be the primary treatment for prevention of life threatening arrhythmias in the majority of patients. Other therapeutic options include pacemakers, implantable cardioverter defibrillators, left cardiac sympathetic denervation, sodium channel blocking medications and lifestyle modification.

## INTRODUCTION

**Figure 1:**
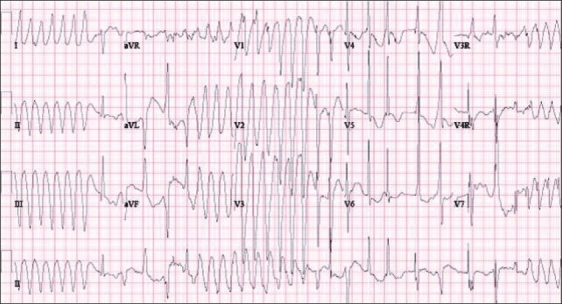
Surface ECG showing torsades de pointes

The congenital long QT syndrome (LQTS) is an inherited channelopathy characterized by prolongation of the QT interval on the surface electrocardiogram and by the occurrence of life-threatening ventricular arrhythmias.[1] The estimated prevalence of this disorder is between 1:5,000 and 1:2,500. Heterogeneous repolarization and early after depolarizations (EAD) cause the characteristic polymorphic ventricular tachycardia, torsades de Pointes (TdP) [Figure 1] and ventricular fibrillation. These arrhythmias result in symptoms such as syncope, convulsions and sudden cardiac death (SCD). The mean age of onset of symptoms is 12 years and earlier onset is usually associated with a more severe form of the disease.[2]

In recent years, there has been a rapid increase in the understanding of the genetic basis of this disease. LQTS is now recognized as a cardiac channelopathy with the identification of over ten genes to date responsible for this disease [Table 1]. It has become apparent that phenotypic presentations, arrhythmia triggers and efficacy of therapy are related to the underlying genotype.[3–5]

**Table 1:** Long QT syndrome genotypes

LQT type	Gene	Function and abnormality
LQT1	KCNQ1	I_Ks_* ↓
LQT2	KCNH2	I_Kr_† ↓
LQT3	SCN5A	I_Na_‡ ↑
LQT4	ANKB	I_Na,K_ ↓ I_NCX_§ ↓
LQT5	KCNE1	I_Ks_ ↓
LQT6	KCNE2	I_Kr_ ↓
LQT7	KCNJ2	I_K1_ ↓
LQT8	CACNA1C	I_Ca-L_‖ ↑
LQT9	CAV3	I_Na_ ↑
LQT10	SCN4B	I_Na_ ↑

* I_Ks_ -Slow component of delayed rectifier current

† I_Kr_ -Rapid component of delayed rectifier current

‡ I_Na_ -Inward sodium current

§ I_NCX_: Na^+^/Ca^+^ exchange current

‖ I_Ca-L_: L type Ca^+^ current

## MATERIALS AND METHODS

A review of the literature was conducted using key words to search PubMed for pertinent articles related to the topics of LQTS, TdP, and their respective acute and chronic therapies. The data was reviewed and relevant information was synthesized and included in the body of this article.

### Diagnostic tests for LQTS

Traditionally, the diagnosis of LQTS has been based on a set of scored clinical criteria known as the Schwartz score.[6] Central to the Schwartz score is the calculated heart rate-corrected QT interval (QTc) from the 12-lead ECG. In addition, the score considers clinical symptoms and family history. A Schwartz score equal to or exceeding 4 indicates high probability of LQTS, whereas a score less than one renders a low-probability LQTS.

Additional clinical tests have shown some value in assisting in the diagnosis of LQTS especially in individuals with a “borderline QTc”. The epinephrine QT stress test, in particular, may be useful in unmasking some types of LQTS, particularly type 1 LQTS (LQT1).[7] More recently, I_kr_ channel blockade using intravenous Erythromycin has been used to unmask type 2 LQTS (LQT2).[8] Because of the limited availability of more definitive tests, the ECG remains one of the most important clinical investigations in the evaluation of LQTS. Investigators have used a cut off value of QTc >0.46 seconds as well as 0.44 seconds for making the diagnosis of long QT syndrome.[6] Both cut off values pose significant challenges. When the QTc measurement approaches these cut off values, it is referred to as a “borderline” QTc. A number of individuals with LQTS have “concealed” LQTS, with QTc values that cross into the normal range (< cut off value). There is a substantial overlap between approximately 10% to 15% of normal individuals and the subset of patients having “concealed” LQTS.[9] These cases of “concealed” LQTS would be missed by relying too heavily on the ECG. On the other hand, many patients could be diagnosed inaccurately with LQTS if the diagnosis was made principally because of a borderline QTc measurement. Age and gender differences also affect QTc values with a tendency for females to have longer QTc measurements.

Genetic testing is commercially available for the identification of the three most common forms of LQTS: LQTS1, LQTS2 and LQTS3. The diagnostic yield of genetic testing is 60-70%.[3,10] Negative genetic testing does not rule out the disease. Genetic testing has become an important diagnostic tool in the evaluation of LQTS as it can further assist in differentiating between normal individuals with a “borderline QTc” and “concealed” LQTS. It can also help in identification of affected family members of probands who are at risk but have remained asymptomatic. Furthermore, genetic testing has allowed clinicians to tailor therapy that is best suited for the particular genotype.

#### LQT1

**Figure 2:**
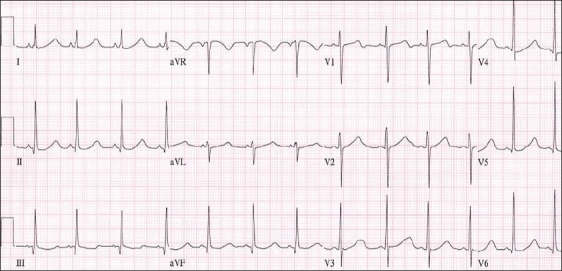
Surface ECG showing prolonged QTc interval with broad based T waves often seen in LQT1

LQT1 is the result of a mutation in the KCNQ1 gene which codes for the α subunit of the I_Ks_ channel. Mutations in this gene account for 45–50% of all LQTS. The classic change on the surface ECG is a broad-based T wave [Figure 2]. In children the findings can be less typical and are more characterized by a long early repolarization phase from T wave onset to peak.[3] The triggers typically associated with LQT1 are related to adrenergic stimulation such as exercise, especially swimming, most likely due to the impaired effects of adrenergic stimulation on the abnormal IK_s_ channel.[5] Therefore, limiting these activities may be appropriate to avoid precipitating arrhythmia.

#### LQT2

**Figure 3:**
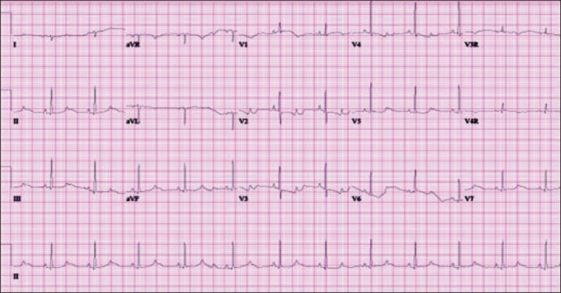
Surface ECG showing prolonged QTc with relatively low amplitude notched T waves seen in LQT2

LQT2 is the result of a mutation in the KCNH2 gene which encodes a subunit of the I_Kr_ channel which accounts for another 35–40 % of LQTS patients. The classic ECG finding for this genotype is low amplitude T waves with prominent notching [Figure 3]. Again, in children, the findings can be more atypical and may be associated with a long late repolarization with an increase in the T wave from peak to termination.[3] The triggers for this mutation are classically associated with sudden auditory stimuli, such as a door slamming shut or an alarm clock.[5] Therefore, eliminating these stimuli as much as possible may be a reasonable precaution for LQT2 patients.

**Table 2:** Common genotypes, triggers and directed therapies

Genetoype	Triggers	Genotype directed therapy
LQT1	- exercise	-avoid competitive and exertional sports
	- tachycardia from other causes	-high efficacy of beta blockers therapy
LQT2	- exercise	-remove clocks, telephones etc. from bed room
	- auditory stimuli	
		-avoid sudden loud noises such as fire drills in school
		-avoid competitive and exertional sports
		-moderate efficacy of beta blocker therapy
LQT3	- bradycardia	-strict restriction from sports may not be necessary
	- rest/sleep	
		-beta blockers may not be efficacious
		-consider adding flecainide or mexiletine
		-consider ICD

#### LQT3

**Figure 4:**
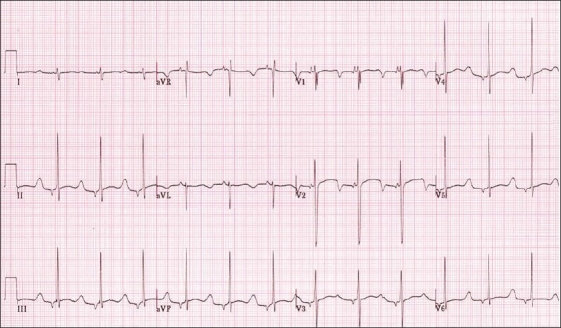
Surface ECG showing prolonged QTc with an isoelectric ST segment and peaked T waves seen in LQT3, also shows a low atrial rhythm

LQT3 is due to a mutation in the SCN5A gene which encodes the I_Na_ and accounts for eight to ten percent of cases. The ECG typically has a long isoelectric ST segment with a peaked T wave Figure 4. Tall, narrow, peaked T waves are more typical to see in children.[3] They are at greater risk for cardiac events at times of slower heart rate, such as during sleep.[5]

All the other mutations make up the remaining one to two percent of the population with LQTS. They are all channelopathies with the exception of LQT4, which is a mutation in a protein that helps to organize important pumps and receptors in the cell membrane key to repolarization.[3] Given the rarity of these mutations, they will not be specifically discussed in regards to therapeutic interventions.

### Acute Therapy of Ventricular Arrhythmias in LQTS

Regardless of the underlying genotype, TdP is the rhythm that is characteristic of acute events in all types of LQTS. This polymorphic form of ventricular tachycardia (VT) is characterized by a twisting of the QRS axis. While it can be a self-terminating rhythm, it can recur and even degrade into uniform sustained VT or ventricular fibrillation (VF). It is these rhythm disturbances that result in syncope or convulsions when self-terminated or sudden cardiac death when persistent.

### Acute Management of Torsades de pointes

The patient with TdP who is in extremis should be treated with direct current electrical cardioversion or defibrillation. Cardiopulmonary resuscitation should be started immediately and the advanced cardiac life support (ACLS) protocol for VT should be instituted.[11]

If the patient is hemodynamically stable or in the event that external cardioversion or defibrillation has failed, the following treatment protocol can be used:

- Magnesium sulfate in a dose of 20–50 mg/kg intravenously initially up to two grams.
- Treat hypokalemia if it is associated with the arrhythmia.
- Shortening the action potential decreases the likelihood of immediate recurrence of TdP. Pacing or administration of isoproterenol to achieve a rate of 90–100 bpm is effective.
  Pacing up to rates of 150 bpm may prevent the ventricular pauses that allow TdP to originate.
  If overdrive pacing can not be performed Isoproternol infusion at a rate of 0.01-2 mcg/kg/minute can be titrated to heart rate effect.
- Adrenergic blockade may be useful in termination of TdP. Short-acting intravenous beta-blockers such as esmolol can be used. Esmolol is given in a loading dose of 0.5 mg/kg intravenously followed by infusion of 100–300 mcg/kg/minute. Alternatively, long acting beta blocker such as intravenous propranolol 0.01-0.1 mg/kg can be given.
- Other anti-arrhythmics that can be tried when aforementioned therapies are ineffective are intravenous phenytoin (Dilantin), intravenous lidocaine and/or intravenous verapamil.
- Withdraw all QT-prolonging drugs.

#### Magnesium sulfate

Magnesium is the drug of choice in TdP accompanying both inherited and acquired LQTS. It is usually very effective, even in the patient with a normal magnesium level.[12–14] Magnesium sulfate administered I.V. is effective in arrhythmias due to early or late after depolarization induced triggered activity rather than any direct effect on repolarization.

#### Isoproterenol

Isoproterenol should only be used when the underlying rhythm is slow, TdP is clearly pause-dependent, and transvenous pacing cannot be immediately implemented. This medication is typically used to prevent recurrence of TdP in acquired LQTS. However, up to one sixth of all patients with acquired LQTS may also have congenital LQTS.[15] Additionally, isoproterenol might have some benefit for patients whose underlying genetic defect has been identified. In animal models beta-adrenergic stimulation by isoproterenol induces TdP by increasing transmural dispersion of repolarization in LQT1 and LQT2 but suppresses TdP by decreasing dispersion in LQT3.[16]

#### Beta- adrenergic receptor blockers

Esmolol is a cardio-selective beta blocker that can be administered intravenously with the added benefits of a short half-life and rapid onset of action for control of TdP.[17] If there is no improvement in the arrhythmia or an adverse reaction occurs, it can be discontinued without sustained sequelae. Intravenous propranolol can also be used but its half life and duration of action is much longer.

#### Lidocaine

Lidocaine is a Class IB sodium channel blocker that has been used to treat pause dependent TdP after myocardial infarction.[18] It may also be useful in LQTS. The loading dose of lidocaine is 1 mg/kg I.V. It can be followed by a maintenance infusion of 20–50 mcg/kg/min. The presumed mechanism of action is a decrease in the depolarization sodium current and suppression of early after depolarization.[19]

#### Phenytoin

Similar to lidocaine, phenytoin is also a Class IB sodium channel blocker with a similar action on phase 0 of the action potential. It can be administered by a parenteral or an oral route. It can be used when TdP is refractory to conventional anti-arrhythmic medications. The I.V. anti-arrhythmic dose is 1.25 mg/kg every five minutes until a desired effect is achieved not to exceed a maximum of 15 mg/kg.

#### Calcium channel blocker

Calcium channel blockade using verapamil can be tried when TdP is refractory to conventional anti-arrhythmic medications. It can be given slowly in a dose of 0.1-0.2 mg/kg I.V. Verapamil is contraindicated in very young children and it should only be used if TdP is unmanageable. Its mechanism of action is related to reducing early after depolarizations induced by abnormal L-type calcium channels and decreasing transmural dispersion of repolarization.[20]

### Prophylactic Therapy

While acute therapy is directed at termination of a potentially life-threatening rhythm, the goal of chronic therapy is prevention, whether primary or secondary, of arrhythmias. Multiple therapeutic modalities currently exist; medical, device based and surgical, for treatment of patients with LQTS.

#### Medications

##### Beta-adrenergic receptor blockers

Beta blockers are the first line therapy for LQTS. Beta blockers are useful for minimizing the adrenergic stimulation that results in rapid changes in heart rate with dispersion of repolarization across the myocardium and after depolarizations that are believed to be the inciting events for TdP. Some reports have documented significant shortening of the QTc[4,21,22] while others have not.[23,24] The efficacy of beta blockers appears to possibly be separate from the effect on QTc itself. QT hysteresis from altered QT adaptation to changes in heart rate has been noted in patients with LQTS. Beta blockers reduce QT hysteresis to values seen in normal patients. Also, beta blockers decrease dispersion of repolarization[21–23,25] and may normalize response of repolarization to adrenergic stimulation such as exercise.[24–26] Beta blockers also attenuate the beta receptor mediated enhancement of L type Calcium channels and restore balance of cardiac ion channel forces.[27]

Despite these salutary effects, beta blockers are not completely protective in preventing cardiac events (syncope, aborted cardiac arrest, sudden cardiac death) in LQTS. In a large retrospective study by Moss *et al*, there was a significant reduction in the rate of cardiac events among all LQTS patients after beta blocker therapy including both probands and family members. However, in patients who were previously symptomatic, 32% of patients had a recurrence in symptoms within five years despite beta blocker therapy. Risk factors for failure of beta blocker therapy include noncompliance, symptoms prior to therapy, younger age, longer baseline QTc (>500 msec), and a genotype other than LQT1.[4,22,27,28]

Patients with LQT1 benefit the most from beta blocker therapy when compared to those with LQT2 and LQT3. One study showed that only 10% LQT1 of patients had breakthrough symptoms while 23% of LQT2 and 32% of LQT3 patients had breakthrough.[28] There is now growing evidence that LQT3 patients are not as well protected by beta blocker therapy as patients with the other forms of LQTS. When prescribing beta blockers, it is important to consider the type, total dose, dosing schedule and adverse effects. The most common beta blockers prescribed are propranolol, atenolol, metoprolol and nadolol. Small studies have suggested that a more cardio selective beta blocker, such as atenolol, may be less efficacious than non-selective beta blocker such as propranolol in treatment of symptomatic patients with LQTS.[27] Observational studies have suggested that α-1-adrenergic receptors may also play an important role in precipitating TdP and beta blockers with alpha-1-blockade should be utilized.[29] Propranolol and nadolol are non selective beta blockers with β1, β2 and α receptor blocking properties where as atenolol and metoprolol are cardioselective β1 receptor blockers. Propranolol is well absorbed orally but has low bio-availability due to first pass hepatic metabolism and requires frequent dosing (three or four times a day) to maintain therapeutic levels unless a long acting preparation is used. Atenolol is commonly used perhaps because of its popularity as a once a day drug due to its longer plasma half life of 6-9 hours. However, this once a day regimen may not be enough to maintain adequate steady state levels throughout the day and should probably be prescribed as a twice a day regimen. Nadolol may prove to be a better once-a-day agent due to its prolonged plasma half life of 10-24 hours but our current practice is to administer Nadolol twice a day taking into account variable metabolism. Adequacy of beta blockade can be evaluated by exercise testing to demonstrate blunting of hear rate response to maximal exercise. Our practice is to obtain an approximately 20% reduction in maximum heart rate (as compared to pre-beta blocker therapy) in patients who are at an age at which formal exercise testing can be performed. Alternatively, peak and trough serum propranol levels can also be obtained. In medically compliant patients with breakthrough symptoms despite an adequate beta blocker dosing regimen, testing should be performed to ascertain if they are “rapid metabolizers.”

##### Mexiletine

Mexiletine is a class IB antiarrhythmic agent with sodium channel blocking properties. In the SCN5A genotype (LQT3), mexiletine has been shown to prevent repetitive sodium channel opening in experimental models.[30] Schwartz *et al*, demonstrated that mexiletine shortens the QT interval in patients with LQT3 and this QT shortening may bear clinical significance.[31] A higher incidence of breakthrough events with beta blocker therapy have been noted in patients with LQT3, as noted above, but there is no consensus that mexiletine should replace beta blockers as the primary therapy in this cohort. Some investigators recommend assessing QT shortening with oral mexiletine and if shortening > 50 msec is seen, mexiletine should be added to beta blocker therapy.

##### Flecainide

Flecainide is a class IC anti-arrhythmic agent which blocks the open sodium channel. It causes slowing of conduction in fast response cells. In patients with LQT3, normalization of the QT interval with short term use of flecainide has been noted.[32] Two studies examined the effects of long-term flecainide therapy and showed that these effects were maintained over follow-up period ranging from 6 to 17 months.[33,34] In adults, dose ranging from 50 mg to 150 mg every 12 hours[32,33] and in children a dose of 50 mg/m^2^ every 12 hours was used.[32] Moss *et al*, assessed the safety and efficacy of flecainide in a small number of LQT3 subjects in a randomized, double-blind, placebo-controlled clinical trial and found significant QTc shortening with no associated adverse reactions.[34] Flecainide has also been useful in treatment of ventricular arrhythmias in the Andersen-Tawil syndrome (LQT7).[35,36] While these results are promising there have been no studies demonstrating clinical efficacy to date for the prevention of symptoms or SCD. At this time flecainide should continue to be considered an experimental modality for LQT3 and may be used as an adjunct to beta blocker therapy.

##### Calcium Channel Blockers

There is experimental evidence that calcium channel blockers decrease the incidence of EAD as well as normalize the dispersion of repolarization and decrease the incidence of ventricular tachycardia.[20,37] Some of these mechanisms of action are similar to beta blockers and may prove to be useful in conjunction with beta blockers. In a case report, the usefulness of verapamil in the treatment of ventricular arrhythmias in Timothy syndrome (LQT8) has been documented.[38] In an intact heart model of LQT3, verapamil has been shown to prevent Tdp by reduction of transmural dispersion of repolarization and suppression of EAD.[39]

##### Potassium supplementation

It has been hypothesized that increasing serum potassium within the physiologic range may augment the repolarizing current and result in improvements in repolarization parameters in individuals with HERG mutations. Increasing the serum potassium level with oral potassium and/or spironolactone has resulted in shortening of the QT interval, QT dispersion and improvement in T wave morphology in patients with LQT2.[40] Whether elevated serum potassium levels can be maintained long-term by these methods is uncertain. Further studies are warranted to evaluate if this will reduce the incidence of life-threatening events in LQTS patients.

##### Potassium channel activators

Potassium channel activators such as nicorandil, pinacidil and cromakalim have been shown to be effective in suppressing ventricular arrhythmias in LQTS in some studies.[41–43] Their long-term effects have not been evaluated.

#### Permanent pacing

Permanent pacing is a consideration for those with persistent symptoms despite adequate medical therapy. Beta blockade is the first line therapy but a side-effect of this therapy is an increased incidence of sinus bradycardia and sinus pauses that prolong repolarization which may induce pause dependant after-depolarizations and precipitate TdP. Pacing is a useful modality to help counteract these effects of beta blockers while allowing treatment with these medications to continue.[44,45] The modality of pacing that is most beneficial is not determined from the studies but the majority of patients had either atrial or dual chamber pacemakers. Despite the improvement in symptoms there is still a significant incidence of SCD in these higher risk patients. In the current era, it makes more sense to implant a dual chamber implantable cardioverter defibrillator (ICD) in high risk patients. This would allow for atrial pacing for those with sinus bradycardia or sinus pauses as a side effect of medical therapy in addition to placement of an ICD as a single procedure given the continued significant risk of SCD. An exception would be symptomatic neonates and infants in whom pacing allows complete beta blockade and may postpone the need for an ICD which can be a complex undertaking in very young patients.

#### ICD

The efficacy and safety of ICDs in patients with LQTS have been shown in several studies but the co-morbidity cannot be ignored, especially in children. Both inappropriate and appropriate shocks may produce severe emotional distress that may trigger additional arrhythmic events and ICD shocks causing an electrical storm. Monnig *et al*, reported an electrical storm in 18.5% of their cohort of patients with LQTS and ICD.[46] ICDs may also be associated with procedural complications, programming challenges, manufacture problems and high cost. Despite these potential difficulties, the ICD offers the most successful therapy for prevention of sudden cardiac death in high risk patients with LQTS.[47–50] It is important to remember that ICDs are not a substitute for beta blockers which remain the primary treatment for LQTS.

##### Recommendations for ICD:[51]

###### Class I

-ICD along with use of beta blockers in patients who have had an aborted cardiac arrest (level of evidence A)

###### Class IIa

-ICD with continued use of beta blockers in patients who remain symptomatic and/or experience VT while receiving beta blockers (level of evidence B)

###### Class IIb

-ICD with use of beta blockers may be considered for primary prevention of SCD for patients in categories associated with higher risk such as LQT2 and LQT3 (level of evidence B)

##### ICD programming challenges in LQTS

TdP accounts for majority of ventricular arrhythmias in patients with LQTS and in many cases this arrhythmia terminates spontaneously. It would be ideal to program the ICD to allow for spontaneous termination of TdP but before any hemodynamic compromise and for the ICD to deliver a shock only for sustained fast VT or VF. One strategy is to program longer VT/VF detection times. Recent ICDs have a non-committed first shock in the VF zone and this will automatically abort the shock if the arrhythmia terminated during ICD charging. Every effort must be made to program the ICD to reduce appropriate and inappropriate shocks. This includes continuing beta blocker treatment, programming VT/VF detection rates higher than exercise stress test derived maximum heart rates, using supraventricular tachycardia discriminators and using dual chamber pacemakers when possible for antibradycardia pacing and rate smoothing.

#### Left cervicothoracic sympathectomy (LCS)

LCS should be considered in patients who have persistent symptoms or receive repeated appropriate ICD shocks despite optimal medical management. This procedure should also be considered in very young high risk LQTS patients who are not ideal candidates for an ICD. This is a surgical procedure in which the lower portion of the dominant left stellate ganglion as well as thoracic ganglia T2 - T4 are ablated. This effectively removes the sympathetic innervation of the heart. The main complications associated with this procedure are Horner's syndrome (90%) and hemidiaphragmatic paralysis (60%).[52] The majority of patients had complete resolution or minimal residual Horner's syndrome and no patients had permanent hemidiaphragmatic paralysis.[52,53] This can be successfully performed with a minimally invasive technique via a thorascopic approach.[54,55] The two largest international studies by Schwartz *et al*, have demonstrated significant reduction in symptoms and ICD discharges.[52,55] There is also a significant decrease in the QTc noted after LCS.[52,53,56] However, LCS is not entirely effective in preventing sudden cardiac death. Among genotyped LQTS patients, LCS tended towards being significantly more effective for those with LQT1 and LQT3.[56]

#### Sports and LQTS

Beyond medical and surgical interventions certain lifestyle modifications are also recommended. Several professional groups have made recommendations for limiting sports involvement. The 36^th^Bethesda Conference published recommendations that asymptomatic patients with baseline QTc prolongation (males with a QTc > 470 msec and females with a QTc > 480 msec) should be limited to low physical intensity competitive sports.[57] The restriction may be liberalized for the asymptomatic patient with genetically proven LQT3. Patients with “concealed LQTS” may be allowed to participate in competitive sports since there is no compelling data available to justify restricting these individuals. LQT1 patients should refrain from competitive swimming. In terms of recreational sports (non competitive), patients with LQTS are generally advised to refrain from high intensity sports such as basketball, football, soccer, singles tennis, weight lifting etc. but may participate in low and moderate intensity activities to be assessed on an individual basis. Patients with ICDs are generally advised not to participate in contact sports and restricted to low physical intensity sports but more data is needed to support this recommendation. From a practical standpoint, these guidelines need to be fully discussed with the patient and family members and a reasonable recommendation for sports participation needs to be made on a case by case basis such that the young patient is also able to enjoy a good quality of life. Overly strict restrictions may result in clinical depression and psychological maladjustments whereas too liberal an approach may put the patient at risk. For young patients and their families who insist on sports participation, we have worked with the families to provide a “safety net”. An emergency action plan is established that includes training parents and anticipated responders in cardiopulmonary resuscitation and automatic external defibrillator (AED) use, access to an AED for early defibrillation and practice and review of the response plan.

#### Avoiding QT prolonging medications

Educating patients (and their families) to avoid QT prolonging drugs is crucial in the management of LQTS. Patients with LQTS who have been asymptomatic for a long period of time can suddenly become symptomatic secondary to using a QT prolonging medication. Commonly used drugs that can prolong the QT interval are erythromycin, ketoconazole, albuterol, epinephrine, ephedrine, nortryptyline etc. A complete list of these medications can be found online at http://long-qt-syndrome.com/lqts_drugs.html as well as www.arizonacert.org. List of drugs to avoid should be provided to families as well as the patient's primary medical doctor.

#### Management Strategies

##### Symptomatic patients

All patients should be started on beta blockers, preferably with a non-selective beta blocker such as propranolol or nadolol with a dose titrated to heart rate effect. If an adequate dose results in significant sinus bradycardia or sinus pauses permanent pacing or placement of a dual chamber ICD can be considered. In patients who have had an aborted cardiac arrest an ICD should be placed. Patients who continue to be symptomatic on beta blockers are also candidates for an ICD. LCS should also be considered if symptoms continue on beta blockers as well as in individuals with an ICD who are experiencing electrical storms. If an ICD is not feasible due to extremely young age, a permanent pacemaker can be implanted to prevent bradycardia and pause dependent arrhythmias. In genotyped patients with LQT3 there is no clinical data that beta blockers should be withheld. Until further clinical data is published, our practice is to continue beta blockers along with mexiletine or flecainide which may be used as adjunct therapy.

#### Asymptomatic patients

Asymptomatic patients are the most challenging population. There are a number of variables to consider when making the decision on how to treat these patients given the complex interactions between genotype, gender, length of QTc and age. The highest risk associated with QTc is seen in those with intervals > 500 msec.[58–60] Given these findings, Priori *et al*, put forth a risk stratification schema for risk of first event before the age of 40 and before therapy. High risk (>50%) included patients with a QTc > 500 msec with LQT1, LQT2 and males with LQT3. Intermediate risk (30-49%) included LQT2 females, LQT3 females with a QTc > 500 msec and both genders with LQT3 with a QTc < 500 msec. Low risk (> 30%) included patients with a QTc < 500 msec who are LQT2 males and both genders with LQT1.[57] Using this risk stratification it would appear reasonable to start all patients with intermediate or high risk on beta blocker therapy. Controversy exists regarding treatment of asymptomatic LQTS patients in the “low risk group” with some investigators opting not to treat these patients. However, this data should be viewed carefully since risk is a “relative” term and an up to 30% probability of a cardiac event does not appear insignificant. Clearly, there are reports of patients with “concealed LQTS” who have experienced sudden cardiac death. Hence, some clinicians such as our group have elected to treat all patients including those in the “low risk” group with prophylactic beta blockers. Patients with the Jervell and Lange-Nielsen syndrome should also be considered high risk even if asymptomatic at the time of evaluation.[61] In genotyped asymptomatic patients with LQT3, mexiletine or flecainide may be used as adjunct therapy to beta blockers.

## CONCLUSION

LQTS is a highly treatable but potentially lethal syndrome. New insights into genotypes and modifier genes will continue to improve our understanding of phenotypic heterogeneity and risk stratification. In an era of genotyping and ICDs, mortality in LQTS should be a rarity rather than a common event. At the present time, beta blockers remain the primary treatment for LQTS and other medications should be considered ancillary therapy.
